# Microtubule Retrograde Motors and Their Role in Retroviral Transport

**DOI:** 10.3390/v12040483

**Published:** 2020-04-24

**Authors:** Gianfranco Pietrantoni, Rodrigo Ibarra-Karmy, Gloria Arriagada

**Affiliations:** Instituto de Ciencias Biomedicas, Facultad de Medicina y Facultad de Ciencias de la Vida, Universidad Andres Bello, Santiago 8370071, Chile; gianfranco.pc@hotmail.com (G.P.); r.ibarrakarmy@gmail.com (R.I.-K.)

**Keywords:** retroviruses, microtubules, dynein, traffic

## Abstract

Following entry into the host cell, retroviruses generate a dsDNA copy of their genomes via reverse transcription, and this viral DNA is subsequently integrated into the chromosomal DNA of the host cell. Before integration can occur, however, retroviral DNA must be transported to the nucleus as part of a ‘preintegration complex’ (PIC). Transporting the PIC through the crowded environment of the cytoplasm is challenging, and retroviruses have evolved different mechanisms to accomplish this feat. Within a eukaryotic cell, microtubules act as the roads, while the microtubule-associated proteins dynein and kinesin are the vehicles that viruses exploit to achieve retrograde and anterograde trafficking. This review will examine the various mechanisms retroviruses have evolved in order to achieve retrograde trafficking, confirming that each retrovirus has its own strategy to functionally subvert microtubule associated proteins.

## 1. Introduction

As obligate intracellular parasites, viruses rely on cellular functions to replicate. A virus will make use of the microtubule network to transport and deliver their genome to the replication site inside a cell, a step that is crucial for the viral replication process, and particularly important for viruses that replicate in the nucleus, such as members of the *retroviridae* family.

Limitations to free diffusion in the cytoplasm have forced viruses to develop efficient mechanisms to subvert the transport systems of the host. Through the use of different microtubule depolymerizing agents, namely, colchicine, vinblastine and nocodazole, it has been shown how relevant microtubule stability is for viral infection [1,2,3]. Many different viruses use the microtubule associated motor proteins to move along microtubules within the cell [1,2,3,4,5]. This review is focused on the mechanism retroviruses use to exploit and subjugate cellular machinery like the microtubule motor dynein for movement. Great advances have been made for HIV-1, but for other retroviruses there have been little advances.

## 2. Microtubules and Their Motor Proteins

Microtubules are the road for the long-distance transport of endocytic/exocytic vesicles, organelles, and viral complexes [2,6,7]. This complex network of tubes is formed by heterodimers of α and β tubulin that interlace in a head-to-tail arrangement forming protofilaments, 13 protofilaments assemble to form a single microtubule filament. A key characteristic of these microtubules is their structural polarity, where one end of the microtubule considered a plus end, can quickly expand or shrink as required (dynamic instability), while the opposite minus end is attached to the microtubule organizing center (MTOC), allowing for directional growth of the microtubule network.

The directional transport that occurs along the microtubules is accomplished by the actions of kinesins and dyneins (Table 1), two ATP-dependent motor proteins. Kinesins are responsible for the transport of cargo within the cell in an anterograde manner, meaning, from the minus to the plus end of the microtubule [8,9], on the other hand, the cytoplasmic dynein motors are in charge of the retrograde transport that occurs, from the plus end towards the MTOC or minus end [10,11]. There are two kinds of dynein, dynein 1 or cytoplasmic dynein, capable of mediating retrograde transport of cargo in the cytoplasm, and dynein 2, also known as axonemal dynein, responsible for the transport in cilia and flagella [7,10,12,13].

A big part of all kinesins transit towards the plus end of the microtubule, nevertheless, there is a small number that moves in the opposite direction [8,9,14]. The kinesin superfamily is composed of a wide variety of different kinesins who vary in shape and form. An archetype of the kinesin family kinesin-1 also known as the “conventional” kinesin, forms heterotetramers composed of a pair of heavy chains (HC) and a pair of light chains (LC). Each of the HC dimers links with two copies of the LC dimer, it is worth noting that despite the fact that HC and LC dimers can be encoded by different genes, each kinesin-1 tetramer is comprised of two identical HC and LC dimers [15]. Cargo carried by kinesins binds directly to specific sites located on the HC part of the kinesin-1 tetramer, this cargo can interact with the motor via tetratricopeptide repeats which are in the C-terminal end of the kinesin LC. Additionally, the expression of several LC kinesin splice variants that possess a different C-terminal domain, will greatly contribute to binding specificity of different types of cargo [9,14,16].

The dynein complex (Table 1) is a minus end-directed motor protein responsible for a wide range of aspects of intracellular motility including retrograde axonal transport [17], organelle transport [10,18], and virus retrograde transport [1,2]. This complex, with a molecular mass of 1.6MDa, is composed of two heavy chains, where several ATPase domains are located, two intermediate chains (IC), three types of light chains (LC) that bind to the IC at different spots, DYNLT, DYNLL and DYNLRB, two additional light intermediate chains (LIC) also bind to the DHC in an autonomous manner. Each of the DHC encompasses six ATPase units (AAA1-6) in tandem, all of them organize in a circular ring shape, that binds to the microtubules thanks to a globular domain that forms at the end of the stalk. In vertebrate organisms, each of the dynein ICs, LICs and LCs are codified by two genes, dynein ICs and LICs may also be alternatively spliced and show different phosphor-isoforms [19,20], the presence of these variations suggests that the different dynein versions are very likely to perform different tasks by binding preferentially to a favored type of cargo. Many studies have also shown that dynein requires other molecules to work in an optimal condition, these accessory proteins which affect dynein’s mechanochemistry such as dynactin, NudEL/NudE, Lis1, and Bicaudal D2, among others, are also implicated to play a role in cargo binding and processivity [21,22,23,24,25,26,27].

## 3. Retrovirus Replication

The retroviral genome consists of two copies of a single stranded positive-sense RNA enclosed in a protein core, the genome contains three main genes *gag, pol* and *env*, that encode the viral proteins. Retroviruses enter cells by binding to specific cellular receptors on the plasma membrane. Then, the virion membrane fuses with the host membrane either at the cell surface or after internalization into endosomes, as a result, the core containing the viral genome and viral enzymes, is delivered into the cytoplasm. In the cytoplasm the core particle becomes the reverse transcription complex as it is exposed to nucleotide triphosphates, then reverse transcription (catalyzed by the reverse transcriptase [RT]) of the RNA genome into double-stranded DNA occurs. The viral DNA must traffic to the nucleus as part of the pre-integration complex (PIC). While the full composition of the different retroviral PICs is still unknown, it is certain that it must at least contain the viral DNA, the integrase (IN) and the capsid (CA) or Gag proteins. After gaining entry to the nucleus, IN permanently integrates the viral DNA into the host genome, after which the integrated provirus is formed [34].

This newly integrated provirus is transcribed into viral RNAs by the action of the host RNA polymerase II and subsequently processed and exported to the cytoplasm. The exported RNA has two possible fates: it can be taken to ribosomes and translated to produce the viral proteins. Alternatively, it can be used to compose the genome of new viral particles. After a virus is fully assembled within the infected cell, it travels to the cell membrane where it buds out, as an immature particle. The maturation occurs in a process triggered by the viral protease [34], where the protease cleaves Gag and Gag-Pol to form the virion. Each phase in this elaborate multi-stage process, from entry to budding, not only requires the aid of multiple host proteins but also the cytoskeleton. In fact, from an infectious retrovirus standpoint, the cytoskeleton of the host cell acts as a pathway that allows the intracellular movement required by a retrovirus for a successful infection.

## 4. HIV-1 and Microtubule Associated Proteins at Early Infection

Retroviruses depend upon microtubule associated dynein for their retrograde transport inside the host cell, were each retrovirus have a particular dynein partner to mediate the movement [19,21,28,29,35,36]. In the last 5 years, most research attention has focused on HIV-1, the causative agent of AIDS, and less advance has been made regarding other retroviruses and their association with the cytoskeleton. Thus, we will first present the history of research into and current understanding of how HIV-1 subverts microtubule associated proteins for efficient retrograde transport to the cell nucleus.

For HIV-1, the first demonstration that retrograde transport of the PIC occurs along microtubules and utilizes dynein comes from the Hope laboratory [23]. This group developed a system that is now widely used to study the intracellular transport of HIV-1: a virion that contains fluorescent Vpr protein and can label the incoming particle, and a fluorescent protein fused to the first 15 amino acids of Scr (S15-fluorescent protein), that is used as a marker of unfused virions. Using these tools, coupled with live imaging, they showed that when anti-IC antibodies were microinjected to cells, viral movement and transport towards the nucleus was halted, but only during the early stages of infection [23]. This raised a question: What cellular partner mediates this directed movement?

In the search for the dynein partner of HIV-1 it was shown that infection can be inhibited by using a dynein light chain peptide belonging to the 19 amino acids of the DYNLL C-terminal [37], but DYNLL did not progress as a relevant factor for HIV-1 infection, although it is essential for other retroviruses (see below). Later, Gallo and Hope reported that the light chain of axonemal dynein, DNAL1, was required for effective HIV-1 infection regardless of viral entry. They also reported that murine leukemia virus (MLV) depends on DNAL1 for infection, though not as prominently as HIV-1 [38]. While a newly arrived HIV-1 particle presents a retrograde net movement, live cell imaging has shown displays of bi-directional movement on microtubules [23,31,39]. In fact, FEZ1, the kinesin-1 heavy chain adaptor, regulates the early transport of incoming viral particles by associating to the HIV-1 capsid [31,40]. This confirmed the tug-of-war model, wherein both anterograde and retrograde transport of cargo takes place, although the net movement is retrograde.

When treating cells with nocodazole, an antineoplastic agent that disrupts dynamic microtubule polymerization, most microtubules will be affected and a small number of microtubules, the stable microtubules, will remain intact. When testing HIV-1 infection after treating cells with nocodazole, infection was not completely abolished [23], this suggested that HIV-1 might use microtubule dependent and independent trafficking for infection, or that HIV-1 was capable of enhancing microtubule stabilization in order to aid its infection process. Microtubule stabilization upon HIV-1 infection was demonstrated by the Nhagavi laboratory [41]. All microtubules contain an end binding protein called EB1 which acts as a stabilizing cap at the end of each microtubule, the Nhagavi laboratory showed that by depleting EB1, the number of stable microtubules and consequently, HIV-1 rate during early infection steps was reduced [41]. They also showed that the EB1-binding protein, Kif4, is targeted by the HIV-1 matrix protein (MA) to induce the stabilization of microtubules through the recruitment of EB-1 to the plus tip of the microtubule [41,42,43]. EB1 recruits other +Tips to stabilized microtubules, among them are the diaphanous related-proteins Dia1 and Dia2. Use of siRNA has shown that both Dia1 and Dia2 coordinate microtubule stability and capsid disassembly, thus regulating uncoating. Silencing of Dia1 or Dia2 reduces HIV-1 infection regardless of the entry mechanism and the cell type; formation of stable microtubules upon HIV-1 infection was also lost, showing that the HIV-1 mechanism of microtubule stabilization is even more complex than previously described [44].

Another protein that participates in microtubule stabilization is microtubule-associated protein 1 (MAP1) [45]. Although not a motor protein, MAP1 has a role in the regulation and cargo-binding of microtubule associated motors [46]. The Arhel laboratory showed that MAP1 can directly interact with HIV-1 p24 capsid protein. Depletion of MAP1 reduced capsid cores association to both stable and dynamic microtubules; the authors suggest that MAP1 might tether capsid cores to the microtubule network, thus promoting cytoplasmic trafficking [47]. These findings confirmed the need of stable microtubules for HIV-1 infection and suggest that MAP1 might provide an anchor point for HIV-1, mediated by p24 at the plus end of the microtubules, before trafficking using dynein activity with an (at that time) unknown partner mediating the association.

It took some time to find a proper partner to pair up HIV-1 PIC with dynein. Two independent articles using different experimental approaches showed that BicaudalD2 (BicD2) was the missing link. BicD2 is a multi-purpose adaptor of dynein that plays a crucial role on development [48,49,50] organelle trafficking [51,52,53] and nuclear positioning; it has also been reported to have important roles in microtubule organization and lipid droplet transport [48,54,55]. It has been suggested that BicD2 functions as a modular link between dynein and the cargo; the N-terminal coiled coil of BicD2 interacts with dynein, whereas the C-terminal coiled coil binds to cargo-specific factors. BicD2 was initially identified as a cellular co-factor for HIV-1 infection in one [56] of the 3 genome-wide screens developed using either shRNA or siRNA [56,57,58] published in 2008. It was not immediately studied, since other proteins seemed to be more attractive at that time for those following the mechanism of the hits described in those screens.

**Figure 1:**
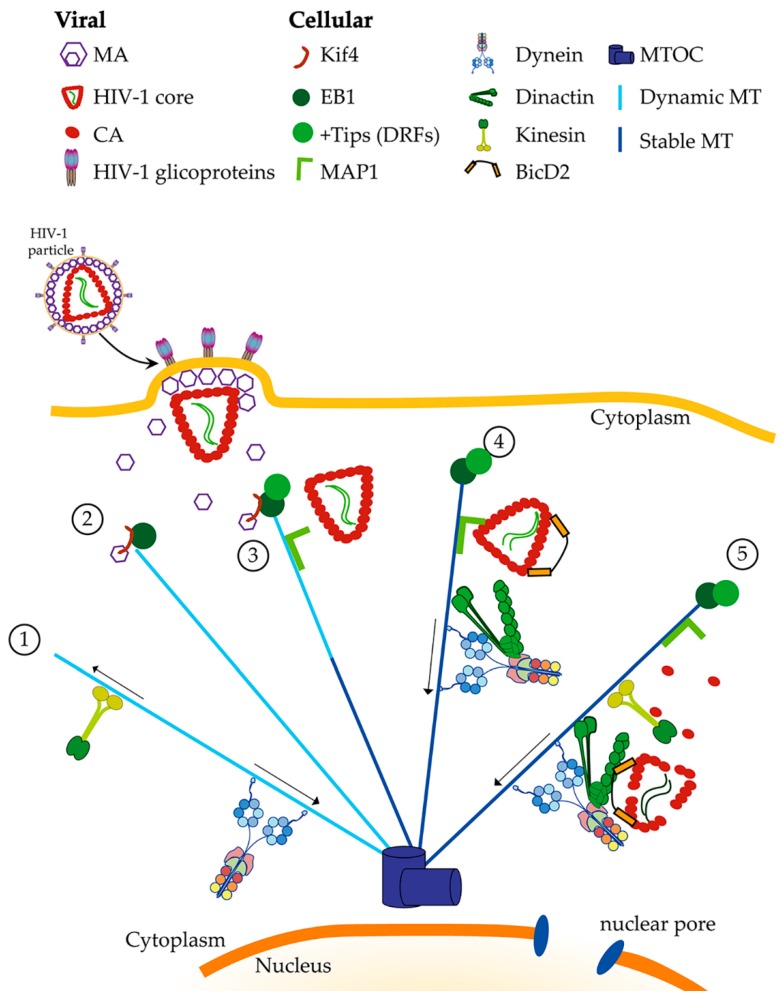
Directed retrograde transport of HIV-1 along microtubules. Directed movement along microtubules is carried out by the ATP-dependent complexes kinesin and dynein, were kinesins are the anterograde motor and dynein is the retrograde motor. This transport can occur along stable and dynamic microtubules. While both kinesin and dynein play crucial roles during retroviral infection, in this figure, we focus on the role of cytoplasmic dynein, leaving kinesins anterograde transport out, as it plays a bigger role during the late stages of the infectious cycle and release of new viral particles (**1**). Once a susceptible cell is infected by HIV-1, fusion of the viral and plasma membrane will occur. Then, the viral core and the matrix protein (MA) are released into the cytoplasm, MA by its interaction with Kif4 will recruit the end binding 1 protein (EB1) to the plus end of microtubules (**2**). This event will allow the stabilization of microtubules and other +Tip, such as diaphanous-related formins (DRF), can be recruited. Microtubule associated proteins, such as MAP1, can be recruited in an HIV-1-independent manner (**3**). HIV-1 core, through the capsid protein (CA), binds to the microtubule stabilization is microtubule-associated protein 1 (MAP1) and Bicaudal D2 (BicD2). It is possible to speculate that MAP1 acts as a hook to attract HIV-1 cores to the microtubules and to the vicinity of dynein (**4**). BicD2 acts as the bridge between HIV-1 core and dynein. A functional HIV-1 transport complex is complete once it contains dynein, dynactin and BicD2 together. Then, a directed retrograde movement towards the microtubule organizing center (MTOC) will occur. Along the movement, reverse transcription and uncoating are simultaneously happening (**5**). Several cellular factors will help along the way to finally cross the nuclear pore and reach the nucleus.

For a while, the research community wondered if indeed a direct interaction with the dynein machinery was occurring, and many, including us, were looking only at dynein and dynactin core components, without much success. In 2017, the Campbell laboratory showed that BicD2 is an essential host factor required for infection [28]. For this, they performed a CRISPR/Cas9 knock out of BicD2 in different cellular models. They showed that BicD2 interacts with the incoming particle and is required for the trafficking of fused particles to the nucleus and for the nuclear import of the viral genome. The direct interaction of BicD2 and CA was shown in vitro using the CA-NC assemblies binding assay and confirmed in infected cells by proximity ligation assay (PLA) [28]. Moreover, they have shown that the KO of BicD2 leads to the expression of interferon stimulated gene (ISG) upon HIV-1 infection, suggesting that in the absence of BicD2, viral DNA accumulates in the cytosol leading to an antiviral response, while in presence of BicD2, the PIC will travel rapidly towards the nucleus along microtubules, and avoid detection by the cellular innate immune machinery. The Aiken laboratory reported a similar finding in early 2018 [29]. Using a different strategy, silencing by siRNAS, they demonstrated that not only BicD2, but also dynactin backbone complex (ACTR1 and DCTN2/DCTN3) facilitate the transport and infection of HIV-1, confirming that BicD2 is the adaptor between HIV-1 PIC and dynein. They also showed that BicD2 is also essential for simian immunodeficiency virus (SIV). Performing biochemical experiments, they delineate the domains of BicD2 that interact with HIV-1 CA. These two studies have finally provided the missing piece of the puzzle: which protein mediated the association of HIV-1 to dynein after the induction of stable microtubules. Now we can envision a model in which, after fusion, MA will recruit EB1 to induce stable microtubules helped by other +Tips, MAP1 will anchor the core to the microtubules and, using BicD2 to piggyback, the core will be transported by dynein to the minus end of microtubules and from there the viral DNA will enter the nucleus (Figure 1).

We have described the process that yield a net movement to the minus end of the microtubules. But as mentioned above, there is indeed a bidirectional movement of the incoming HIV-1 capsid cores, mediated by kinesins. This bi-directional movement is also important for uncoating (loss of capsid). Uncoating is a highly regulated process necessary for nuclear entry, no uncoating or accelerated uncoating are deleterious for infection [32,44,59,60,61]. Although it is not clear how the mature capsid core disassembles to allow the nuclear translocation of the lentiviral genomes, two models have been debated. The first one proposed that capsid remains fully intact until it arrives to the nuclear pore, and its disassembly occurs as the viral DNA is imported into the nucleus. The second model proposes that capsid undergoes structural changes in the cytoplasm resulting in capsid rupture where a portion of capsid remains intact until disassembly is completed at the nuclear pore, this model agrees with the idea that during trafficking along microtubules, cytoplasmic dynein and kinesin 1, particularly Kif5B, are required for this process as the genetic inhibition of DHC and Kif5B by siRNA, or pharmacological inhibition of dynein function, delayed uncoating, and reduced HIV-1 infection [33]. Furthermore, FEZ1 a kinesin adaptor that bridge HIV-1 cores to Kif5B is important for both traffic and uncoating [31,32,33], were phosphorylation of FEZ1 is required to recruit Kif5B to the HIV-1 cores and to mediate uncoating along movement [32]. If FEZ1 and Kif1B promote uncoating and BicD2 protect from accelerated uncoating and/or rapidly move HIV-1 PIC to the nuclear pore area [28], there must be a highly regulated balance that couples trafficking with uncoating to allow sufficient loss of capsid to permit nuclear entry, but a such a rate that viral DNA is not detected by the antiviral machinery in the cytoplasm. Which other players are regulating this process is still a matter of debate.

## 5. BIV, MLV and PFV directly Associate to Dynein Light Chains

Less information is available for other retroviruses, even lentiviruses, which are mostly used for comparison, rather than objects of study. For example, bovine immunodeficiency virus (BIV), requires microtubules and dynein for retrograde transport. The capsid protein (CA) of BIV directly associates to dynein LC DYNLL [19], but it is not known whether BIV can induce microtubule stabilization or if any other component of the retrograde transport machinery or microtubule associated protein is required for infection.

Primate foamy virus (PFV) is a virus from the *spumavirus* genus and the prototypic model for foamy virus replication. This virus also makes use of the cellular motor proteins, before nuclear entry, it first traffics to the microtubule organization center (MTOC) in an event requiring the activity of dynein. This was demonstrated by abolishing dynein activity within cells via overexpressing a dominant negative form of the dynactin component p150^Glued^. The association that occurs between dynein and PFV is triggered by the interaction between the dynein LC DYNLL and Gag [18], the association between the two was demonstrated by co-localization and co-immunoprecipitation assays. These two examples might lead one to think that unlike HIV-1 all other retroviruses associate to dynein by the LC DYNLL, but results of our laboratory tests showed something different [20,21].

Murine leukemia virus (MLV) is one of the most widely used virus models and is also used as a gene vector (both in vitro and in vivo). We have previously demonstrated that the dynein regulator p50/dynamitin, together with the dynein IC, associate with the MLV PIC [20], this led us to discover that dynein regulators p50 and NudEL are essential for MLV infection [20,29]. Dynein IC also plays a crucial role in MLV infection, since the silencing of the IC by shRNA reduced MLV infection, albeit not as strikingly as p50 or NudEL knock-downs [20]. Dynein IC is important for associating dynein with cargo and is essential to anchor LCs to the complex. The latter finding led us to study the role of LCs on MLV infection. Our research demonstrated that silencing of the LC DYNLRB2 reduced MLV infection. On the other hand, over-expression of the DYNLRB2 levels had an enhancing effect over MLV infection, showing a functional role for DYNLRB2 on the MLV infection process [21]. Even though there has been a lack of evidence for a direct physical interaction between the MLV proteins and DYNLRB2 and a clear role on MLV traffic along microtubules, tools such as fluorescent label virions were p12 is tagged with GFP [22] will allow us to further confirm the essential role of dynein and its LC DYNLRB2 in MLV early infection. Unpublished work from our group, using GFP-p12-labeled MLV, point to DYNLRB2 being essential for both trafficking MLV along the microtubules and its arrival to the nucleus (manuscript under preparation).

**Figure 2:**
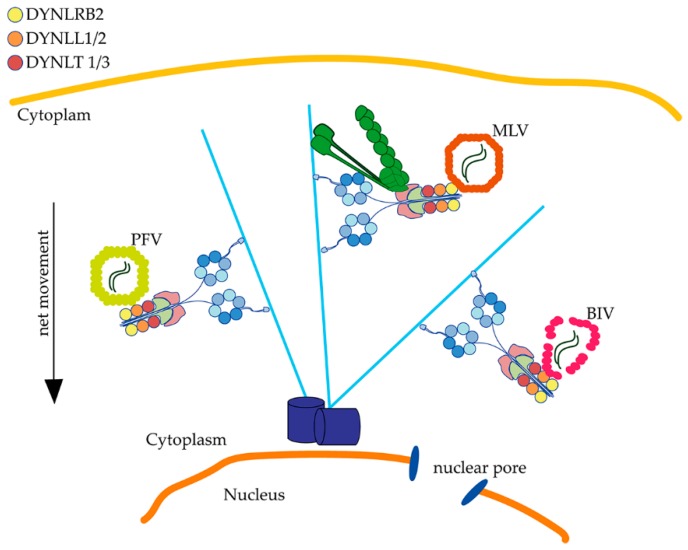
Retroviruses associate with dynein by direct interaction with light chains. Dynein light chains are essential for primate foamy virus (PFV), murine leukemia virus (MLV) and Bovine immunodeficiency virus (BIV) infection. PFV and BIV directly associate through Gag or CA to DYNLL for their retrograde transport. MLV requires dynein and dynactin for infection, and although no physical interaction has been demonstrated, the light chain DYNLRB2 is functionally required for MLV infection.

Several HIV-1-focused reports that used MLV as a control or comparison have shown the requirement for dynein and dynactin activity, but do not agree on the importance of BicD2, which may be dispensable [28] or somehow required [29]. We can conclude that for MLV, BIV and PFV, dynein LC are important for retrograde movement, but direct or functional associations to dynein are mediated by specific LC (Figure 2), and can speculate that if the binding of a retroviral core to dynein is direct, then the induction of stable microtubules is not necessary for infection, since MLV does not induce microtubule stabilization upon infection [41].

## 6. Conclusions

Retroviruses make use of the cellular machinery to achieve effective infection, and the microtubule cytoskeleton, as well as the dynein and kinesin motors, are cellular components that viruses require and are used in many steps of a virus replication cycle. Though the requirement for this machinery during the early events of infection is shared among the retroviruses, each virus appears to use these components in a different way. Whereas HIV-1 subverts the retrograde transport machinery by binding BicD2 for a piggyback ride on dynein, other retroviruses directly associate to dynein components. It is possible to speculate that these mechanistic differences are related to the virus capacity to evade the antiviral machinery on the cell; MLV, for example, does not induce an antiviral state and also retains more CA protein associated to the preintegration complex than HIV-1, since MLV does not have the need to undergo uncoating to fit through the nuclear pore, the structure of the capsid shell might be used to bind directly to dynein components. HIV-1, on the other hand, must go through uncoating, but in a very regulated manner, in order to evade an antiviral response from the host. Thus, while moving along the microtubules, the same proteins that are subverting for trafficking (BicD2 and FEZ1/Kif5B) are helping control the loss of capsid. More research is required to confirm if this hypothesis is true, and the study of other retroviruses must be included.

One can also speculate that older retroviruses were directly interacting with the cellular motors, and as they were evolving, a diversification of the mechanisms with which to accomplish retrograde movement appeared. More research is needed to determine the cytoplasmic transport strategies of other retroviruses, since knowledge of how different retroviruses interact with the cytoskeleton will likely provide important new insights into their biology and evolution.

## Figures and Tables

**Table 1:** Motor proteins and their roles on viral infection.

Structure	Cellular Function	Relevance for Viral Infection
**Dynein** 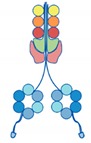	- Participates in retrograde organelle transport inside the cell. - Plays crucial role regulating stages of cell cycle. - Requires other molecules to work optimally that also play a role in cargo binding NudEL/NudE, Lis1, bicaudal D2).	- Participates in virus retrograde transport, carrying viral PIC towards the nucleus [23]. - Interacts with HIV-1 by associating with BicD2 [28,29]. - Interacts directly with BIV capsid for retrograde transport [19].
**Dynactin** 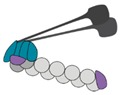	- Cofactor that activates dynein mediates vesicle mobility. - Links dynein to various cargos. - Strengthens dynein binding to microtubules.	- Required for retroviral infection, possibly mediating association to dynein [20,29].
**Kinesin** 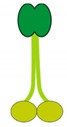	- Participates in anterograde transport within the cell. - Contains a kinesin “tail” where cargo binds and a motor domain responsible for its movement.	- Kinesin 4, 8 and 13 actively regulate microtubule dynamics, aiding in the process of infection [30]. - Kinesin-1 adaptor, FEZ1 has been shown to regulate early transport of viral particles by associating to HIV-1 capsid [31]. - Kinesin might also play a role in uncoating of HIV-1 thanks to FEZ1 and Kif5B [32,33].
